# Site-1 protease function is essential for the generation of antibody secreting cells and reprogramming for secretory activity

**DOI:** 10.1038/s41598-018-32705-7

**Published:** 2018-09-25

**Authors:** Muna Al-Maskari, Matthew A. Care, Emily Robinson, Mario Cocco, Reuben M. Tooze, Gina M. Doody

**Affiliations:** 1 Section of Experimental Haematology, Leeds Institute of Cancer and Pathology, University of Leeds, Leeds, LS9 7TF United Kingdom; 2 Bioinformatics Group, School of Molecular and Cellular Biology, University of Leeds, Leeds, LS2 9JT United Kingdom

## Abstract

The unfolded protein response (UPR) and activation of XBP1 is necessary for high secretory efficiency and functional differentiation of antibody secreting cells (ASCs). The UPR additionally includes a branch in which membrane-bound transcription factors, exemplified by ATF6, undergo intramembrane-proteolysis by the sequential action of site-1 (MBTPS1/S1P) and site-2 proteases (MBTPS2/S2P) and release of the cytoplasmic domain as an active transcription factor. Such regulation is shared with a family of CREB3-related transcription factors and sterol regulatory element-binding proteins (SREBPs). Of these, we identify that the CREB3 family member CREB3L2 is strongly induced and activated during the transition from B-cell to plasma cell state. Inhibition of site-1 protease leads to a profound reduction in plasmablast number linked to induction of autophagy. Plasmablasts generated in the presence of site-1 protease inhibitor segregated into CD38^high^ and CD38^low^ populations, the latter characterized by a marked reduction in the capacity to secrete IgG. Site-1 protease inhibition is accompanied by a distinctive change in gene expression associated with amino acid, steroid and fatty acid synthesis pathways. These results demonstrate that transcriptional control of metabolic programs necessary for secretory activity can be targeted via site-1 protease inhibition during ASC differentiation.

## Introduction

During terminal differentiation of B-cells to plasma cells (PCs), specific gene expression programs are instigated to allow adaptation to the secretion of large amounts of immunoglobulin. A critical role for the transcription factor XBP1 has been identified linking differentiation, ER stress and secretory apparatus expansion^1,2^. The initial data describing a role for XBP1 in PC generation was consistent with the secretion of immunoglobulin triggering an unfolded protein response (UPR)^3^. Later reports suggested that XBP1 could be expressed in cells that did not secrete immunoglobulin, challenging the idea that a UPR is required^4,5^. Furthermore, results from a B-cell conditional knockout demonstrated that XBP1 was not required for the early stages of PC differentiation, but was required for efficient immunoglobulin secretion^6^. These data were additionally corroborated in another model of B-cell specific deletion of XBP1, linking XBP1 to the regulation of ER remodelling required for high rates of secretion^7^. Unlike the proposed requirement for XBP1, available data suggest that both the PERK and ATF6 axes of the UPR may be dispensable for the formation of PCs^8,9^. Collectively, the available evidence suggests that B-cells utilize the UPR in an unconventional fashion^10^, and other components of the ER stress response may provide partially redundant regulation of the secretory apparatus during PC differentiation. Amongst these the CREB family has not been explored in the context of B-cell differentiation.

CREB3L2 is one of 5 members of the CREB3 (CREB-cAMP response element binding protein) family^11^. This is a group of bZIP transcription factor proteins that are synthesized as latent ER resident transmembrane proteins and require protease cleavage in the Golgi to release the active transcription factor component^12^. The CREB3 family members are implicated as evolutionarily conserved regulators of the secretory apparatus and potentially of the UPR. CREB3L2 and other CREB3 family members share their mechanism of activation with ATF6 and sterol regulatory element binding protein, SREBP, a major transcriptional regulator of sterol and lipid synthesis. All of these factors are released from the ER, following appropriate stimulation, and migrate to the Golgi where they are cleaved by the sequential action of S1P and S2P^11,13^. This event releases the cytosolic transcription factor component that migrates to the nucleus and binds to DNA, regulating gene expression.

The sequential process of intramembrane proteolysis controlled by S1P and S2P provides a potential avenue for therapeutic intervention targeting the group of transcription factors sharing this process of regulation. Evaluation of the pathway was originally performed in relation to control of the SREBP in the context of potential control of hepatic lipid synthesis^14^. This led to the development of a selective inhibitor and tool compound for selective dissection of the pathway in cell biology. Here, we describe the progressive accumulation of CREB3L2 during PC differentiation and utilize the selective S1P inhibitor PF-429242 to establish that S1P-regulated events are essential for efficient ASC differentiation and regulation of genes involved in the metabolic pathways necessary for adaptation to antibody secretion. This pathway reinforces the direct link between the secretory apparatus and the establishment of ASC state.

## Results

### CREB3L2 is induced and processed to the active form during PC differentiation

**Figure 1:**
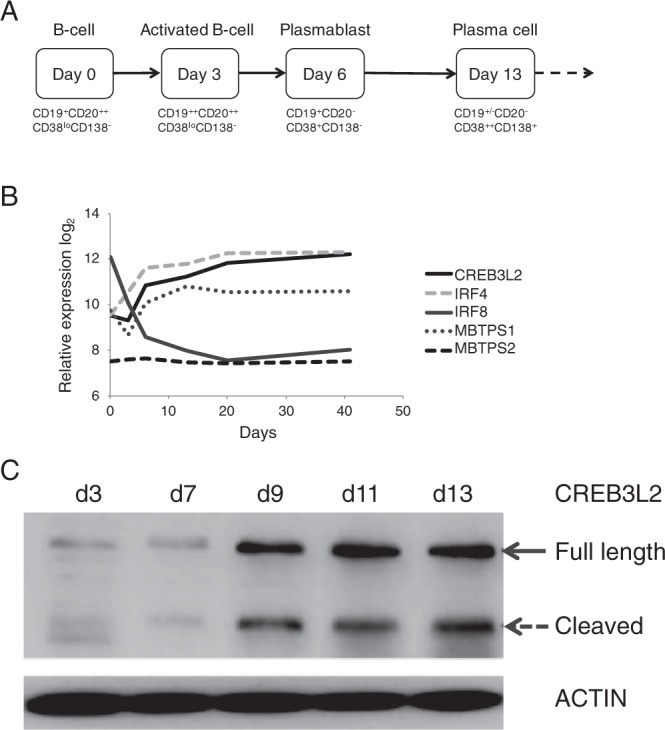
Expression of CREB3L2 in differentiating primary human B-cells. (**A**) Schematic of *in vitro* B-cell differentiation with key stages and phenotypes. (**B**) Relative mRNA expression levels for *IRF4*, *IRF8*, *CREB3L2*, *MBTPS1* and *MBTPS2* during *in vitro* generation of PCs derived from microarray data. (**C**) Protein expression of CREB3L2 during *in vitro* differentiation of primary human B-cells. The day of culture is indicated at the top. Intact and dashed arrows show migration of full-length protein and cleaved C-terminus, respectively.

After appropriate stimulation B-cells undergo a step-wise reprogramming for dedicated antibody secretion. Recent developments of *in vitro* models of human PC differentiation provide the opportunity to dissect the regulatory networks that govern this transition (Fig. 1A)^15–17^. In a detailed characterization of gene expression spanning multiple stages of human PC differentiation, *CREB3L2* was identified as one of the genes mostly strongly induced in PCs, distinct from other CREB3 family members^15^. This pattern of expression, with *CREB3L2* highly expressed in PCs compared to B-cells, is also conserved in mice^18^. Similarly, the level of the *MBTPS1* (S1P) enzyme responsible for the first phase of CREB3L2 processing also increases with differentiation, while the *MBTPS2* (S2P) enzyme remains stable (Fig. 1B). Furthermore, in the gene expression profiling experiments *CREB3L2* has a similar dynamic to other TFs critical in PC differentiation such as *IRF4*. This contrasts sharply for example with the expression of *IRF8*, a negative regulator of PC differentiation (Fig. 1B)^19,20^.

Based on the gene expression data, CREB3L2 is predicted to increase as B-cells transition to antibody secreting cells (ASCs), however generation of the active transcription factor depends on regulated intramembrane proteolysis. To determine whether the protein expression of CREB3L2 correlated with mRNA levels and differentiation stage, human B-cells were isolated from normal donors and differentiated *in vitro*. CREB3L2 expression was evaluated at defined intervals corresponding to activated B-cells/pre-plasmablasts (day 3), plasmablasts (day 6/7) and PCs (day 11+) (Fig. 1C)^15,21^. The results demonstrated the presence of two forms of CREB3L2, with molecular weights consistent with the full-length (80 kDa) and processed form (60 kDa). The molecular weight of the processed form was independently verified by transfection of an expression construct encoding the transcriptionally active N-terminus (data not shown). Both forms were present at each time point, however in activated B-cells (day 3) there is very little processed CREB3L2. Subsequent to this at the plasmablast and PC stages, CREB3L2 full-length and cleaved forms were increasingly expressed. Thus, the protein expression pattern agreed with the observed levels of mRNA, and demonstrated that CREB3L2 is both expressed and processed during PC differentiation.

### S1P inhibitor blocks CREB3L2 processing but does not alter plasmablast to PC maturation

**Figure 2:**
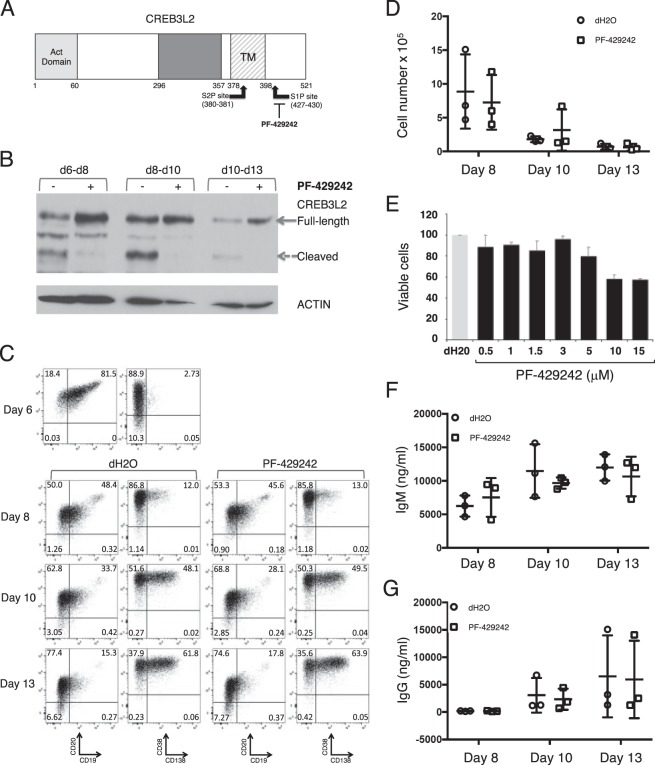
S1P-specific inhibitor PF-429242 blocks CREB3L2 processing. (**A**) Diagram of CREB3L2 structure with proteolytic cleavage sites. Act domain, Activation Domain. bZIP, Basic Leucine Zipper Domain. TM, Transmembrane Domain. (**B**) *In vitro* generated human ASCs were treated with 10 μM PF-429242 or vehicle control (dH2O) on the day that appears first (d6, d8, d10) and protein lysates generated on the day that appears subsequently (d8, d10, d13) were analyzed by Western blotting. Intact and dashed arrows show migration of CREB3L2 full-length protein and cleaved C-terminus, respectively. (**C**) *In vitro* differentiated human B-cells were treated with 10 μM PF-429242 or vehicle control (dH2O) at day 6 and analyzed subsequently for phenotype on the indicated days. Live cells were evaluated for expression of B-cell markers CD19 and CD20 and ASC markers CD38 and CD138. Percentages of cells are shown within the quadrant gates. The experiment was performed with 4 donors, a representative donor is shown. (**D**) Samples treated as in part (**B**) were assessed for cell number using CountBright beads. (**E**) *In vitro* activated human B-cells from 4 donors were treated with indicated amount PF-429242 or vehicle control (dH2O) at day 6 and assessed for cell number at 48 h. Results are displayed as the percentage viable cells relative to control. Supernatants from samples in part (**B**) were analyzed by ELISA for production of (**F**) IgM and (**G**) IgG. Significance was determined by t-test.

The pattern of CREB3L2 expression observed during PC differentiation is consistent with processing via intramembrane proteolysis. CREB3L2 is a single-pass type 2 membrane-spanning protein and its proteloytic cleavage occurs in the Golgi via sequential activities of firstly S1P, in an exposed luminal site, and secondly S2P which catalyses an intramembrane cleavage (Fig. 2A)^22^. The reversible, competitive aminopyrrolidineamide inhibitor for S1P, PF-429242, provides a tool compound with which to dissect the involvement of this pathway of transcription factor regulation^14^.

Since the maximal expression of CREB3L2 was observed at the plasmablast to PC transition we initially tested the impact of PF-429242 treatment during the window of maturation from plasmablast to PC. Indeed, treatment of maturing plasmablasts at each of the time points assessed with a dose of PF-429242 previously demonstrated to inhibit the processing of SREBP^14^ strongly reduced the expression of cleaved CREB3L2. This processing event thus provides an indicator of S1P pathway activity in both plasmablasts and PCs, which can be effectively subject to pharmacological inhibition (Fig. 2B). Additional bands were also observed in the Western blots, lower than the predicted molecular weight of full-length CREB3L2, which are consistent with the previously described non-glycosylated versions of the protein^22^.

Surface phenotype is a key indicator of progression through the differentiation from plasmablast to PCs and can be tracked using the combination of CD19, CD20, CD38 and CD138 (Fig. 1A). To determine whether inhibition of S1P at the plasmablast stage impacted on the acquisition of the PC phenotype, ASCs were treated with PF-429242 or vehicle and analyzed at sequential time points by flow cytometry. However, there were no discernable changes in expression of CD19, CD20, CD38 or CD138 after treatment with the S1P inhibitor (Fig. 2C). Thus although effective in blocking CREB3L2 processing, PF-429242 treatment did not impact on phenotypic changes during ASC differentiation. Furthermore PF-429242 treatment also did not appreciably impact on cell number over a range of doses spanning 0.5 μM to 15 μM (Fig. 2D,E) or secretion of IgM and IgG as assessed from culture supernatant (Fig. 2F,G).

Therefore, despite having a profound effect on CREB3L2 processing, inhibition of S1P with PF-429242 failed to substantially perturb the maturation of plasmablasts to the PC state as assessed by cell counts, surface phenotype and immunoglobulin secretion. This suggests that CREB3L2 or other S1P-regulated transcription factors are not essential for the late stages of ASC differentiation *in vitro*.

### S1P inhibitor prevents generation of ASC from activated B-cells

**Figure 3:**
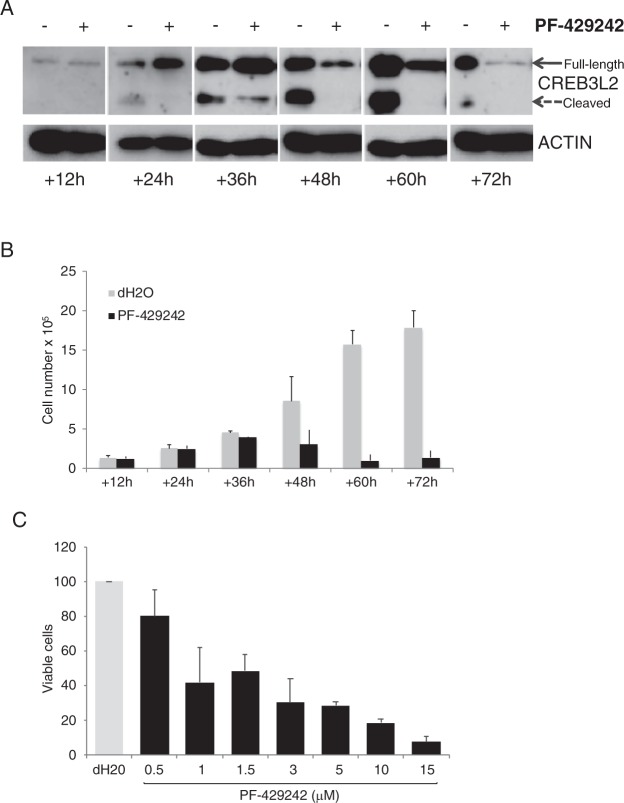
Kinetic and dose analysis of S1P-specific inhibitor PF-429242 during the early phase of ASC generation. (**A**) *In vitro* activated human B-cells were treated with 10 μM PF-429242 or vehicle control (dH2O) at day 3 and assessed for CREB3L2 processing at 12 h intervals. Representative results are shown from a total of 7 donors. (**B**) Samples from part (**A**) were assessed for cell number at the indicated time points. Displayed are the average cell number ± standard deviation derived from 7 donors. (**C**) *In vitro* activated human B-cells from 4 donors were treated with indicated amount PF-429242 or vehicle control (dH2O) at day 3 and assessed for cell number at 48 h. Results are displayed as the percentage viable cells relative to control.

On the other hand, analysis of gene expression in diffuse large B-cell lymphoma has shown that *CREB3L2* is characteristic of the activated B-cell (ABC) subset^23^, and CREB3L2 protein is detectable in primary activated B-cells/pre-plasmablasts in the *vitro* model. At the activated B-cell stage, the cells are undergoing rapid proliferation, with as many as 3 divisions within the space of 24 h^24^. From day 3 to day 6 the cells continue to divide prior to completing differentiation, which is accompanied by exit from cell cycle. Thus, CREB3L2 and the S1P pathway may function in B-cells initiating the process of PC differentiation and during the process of cell proliferation. To address this question, activated B-cells at day 3 of differentiation were treated with PF-429242 or vehicle and samples were collected every 12 h until day 6. In control vehicle treated cells CREB3L2 was evident primarily in unprocessed form at 12 h, by 24 h the processed form was evident and both unprocessed and processed forms accumulated peaking at 60 h (Fig. 3A). Thus in the absence of PF-429242, CREB3L2 is rapidly upregulated and processed as activated B-cells commence the transition to the plasmablast state. Over the time course, inhibition of CREB3L2 cleavage by PF-429242 was accompanied initially by a loss of processed and increased full-length protein, first detected 24 h and maximal 36 h post-treatment. This was followed by a loss of both forms such that by 48 h processed CREB3L2 was not detected and by 72 h PF-429242 had abrogated expression of CREB3L2.

Accompanying the inhibition in first processed and then total CREB3L2, we observed a marked reduction in cell number after exposure to PF-429242. After initial exposure cell numbers expanded equivalently at 12 h, 24 h and 36 h, but from 48 h onward rapidly diverged with progressive loss of cell number in PF-429242 treated conditions (Fig. 3B). The loss of cell viability showed a dose-dependance, with less than 10% of cells surviving at 48 h when treated with 15 μM PF-429242 (Fig. 3C). Thus the impact on population cell number showed a delayed kinetics consistent with the pattern of impact on protein expression.

**Figure 4:**
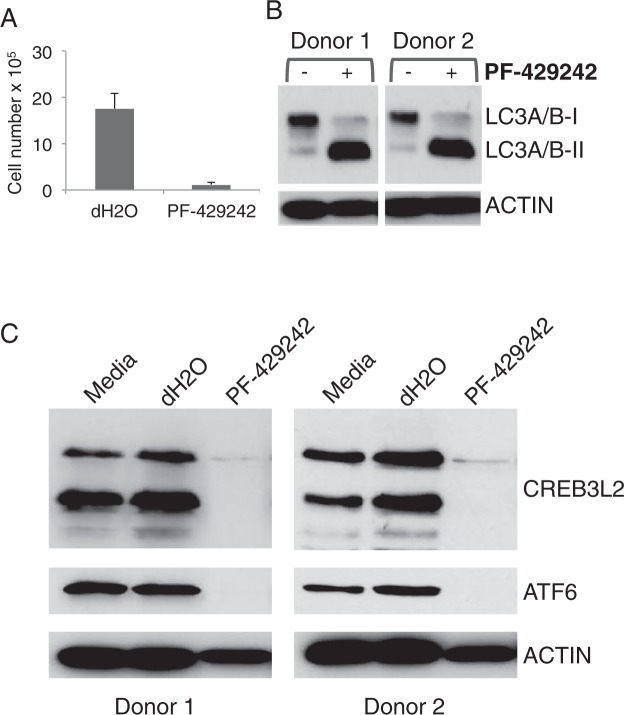
S1P inhibition induces autophagy and affects multiple UPR-related TFs. (**A**) *In vitro* day 3 B-cells were treated with 10 μM PF-429242 or vehicle control (dH2O) for 72 h and were assessed for cell number using CountBright beads. Shown are the average cell numbers from 7 donors ± standard deviations. Protein lysates generated from day 3 cells treated for 72 h with 10 μM PF-429242 were evaluated for (**B**) autophagy markers or (**C**) CREB3L2 and ATF6 by Western blotting. Shown are representative results from 2 donors, a total of 7 donors were evaluated.

The effects on cell number were further corroborated in a number of additional donors as an end-point assay after 3 days of PF-429242 treatment (Fig. 4A). Autophagy provides an adaptive response to misfolded protein in parallel with the UPR and provides a supportive pathway during plasma cell differentiation. Consistent with differentiation under stress and the reduction in cell number, PF-429242 treatment also promoted the accumulation of LC3II relative to LC3I (Fig. 4B). Thus PF-429242 treatment during the activated B-cell to plasmablast transition has profound effects on the efficiency of population expansion and is accompanied by markers of autophagy as an indicator of cellular stress. This contrasts markedly with the lack of effect observed with similar treatment during the subsequent plasmablast to PC transition.

Based on the available data, CREB3L2 is a strong candidate for mediating UPR-associated programs downstream of S1P. However, the generation of active transcription factors by regulated intramembrane proteolysis, and S1P in particular, extends to prototypical sensors of ER stress such as ATF6. To determine whether S1P inhibition similarly affected the other conventional arms of the UPR, lysates were assessed by immunoblotting with antibodies that recognize the active form of ATF6 (Fig. 4C). Consistent with S1P-regulated processing, treatment of differentiating B-cells with PF-429242, led to a loss of detectable ATF6. Thus, although CREB3L2 provides an effective read-out of S1P-pathway inhibition, the profound impact on ASC differentiation mediated by PF-429242 during the activated B-cell to plasmablast transition is likely to involve concerted effects on several transcriptional pathways that share a similar mode of regulation.

### S1P inhibition distorts the plasmablast phenotype and alters secretory potential

**Figure 5:**
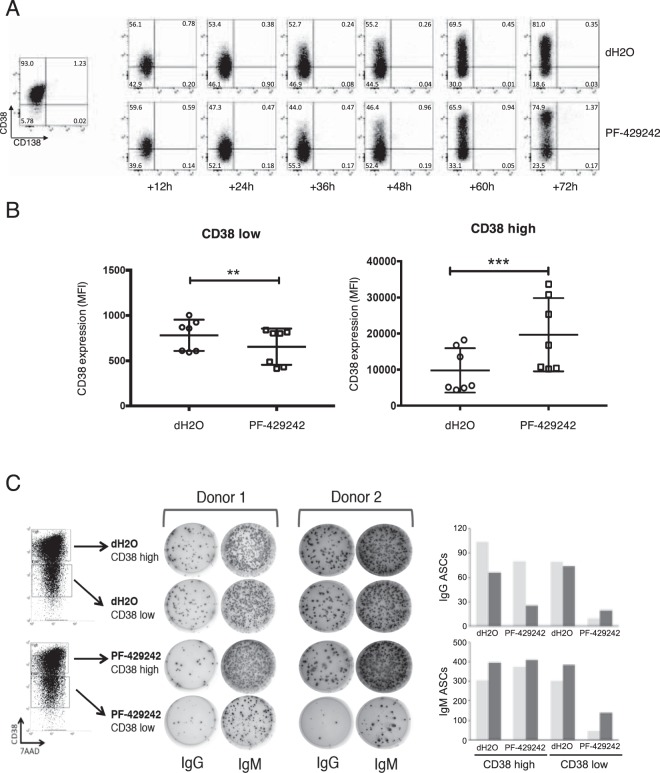
S1P inhibition alters phenotypic maturation and prevents antibody secretion. (**A**) *In vitro* activated human B-cells were treated with 10 μM PF-429242 at day 3 and analyzed at 12 h intervals. Live cells were evaluated for surface phenotype. (**B**) The levels of CD38 expression in the lower left quadrant (CD38 low) or upper left quadrant (CD38 high) were determined for 7 donors at the 72 h time point. Data depict the mean ± standard deviation with significance determined by t-test (**P = 0.003, ***P = 0.001). (**C**) Day 3 human differentiated B-cells were treated with 10 μM PF-429242 or dH2O for 72 h. After the incubation, the cells were sorted based on CD38 (high and low). An equal number of sorted cells (2,000 cells) were used to quantify ASCs using ELISpot for IgM and IgG.

Given the profound impact of S1P inhibitor on the number of plasmablasts generated from activated B-cells, we next examined whether this translated into an effect on phenotypic maturation. During the activated B-cell to plasmablast window, despite the dramatic impact on cell number, the progressive acquisition of CD38 associated with plasmablast/PC differentiation was not abrogated. Consistent with the delayed effects on cell number no phenotypic difference could be identified up to 36 h. From 48 h onward, while acquisition of CD38 expression was observed in PF-429242 treated cells, a separation into two more distinct populations of CD38^high^ and CD38^low^ cells was evident. Indeed, where CD38 was acquired a higher MFI was evident amongst PF-429242 cells (Fig. 5A,B).

Since the defining feature of ASCs is the ability to secrete antibodies, an ELISpot assay was employed to measure Ig production from purified cells. *In vitro* activated B-cells were treated with PF-429242 or vehicle on day 3 for 72 h. On day 6, cells were sorted based on CD38 expression levels (CD38^high^ and CD38^low^) and equal numbers were seeded for IgM and IgG ELISpot (Fig. 5C). In control cultures an equal number of cells from both CD38^high^ and CD38^low^ sorts were capable of secreting IgM, whereas the number of IgG secreting cells was slightly lower in the CD38^low^ population. For PF-429242 treated samples, the number of IgM-secreting cells in the CD38^high^ group was comparable to that observed in the control. In contrast, the number of IgM-secreting cells derived from the CD38^low^ population was reduced by 2-fold or greater. The most profound effect of PF-429242 was on the generation of IgG-secreting cells, such that the number of IgG-producing was lower in both populations but was most reduced amongst the CD38^low^ population (Fig. 5C right panels). Thus, treatment of activated B-cells with PF-429242 led both to a substantial reduction in total cell number and the generation of CD38^low^ cells with a reduced immunoglobulin secretion capacity.

### S1P regulates adaptive metabolic programs required for secretory activity

**Figure 6:**
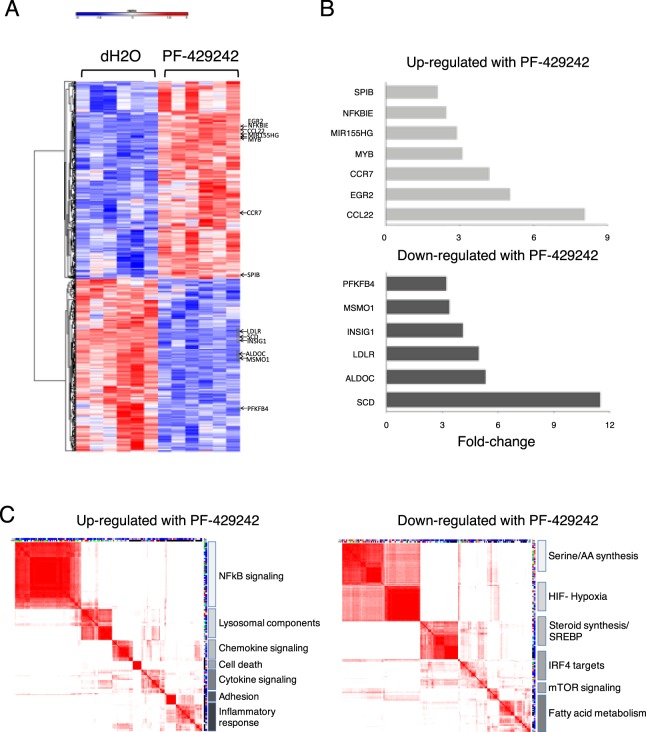
Gene expression analysis identifies distinct gene signature enrichment in PF-429242 treated plasmablasts. (**A**) Day 3 B-cells from 6 donors were treated with 10 μM PF-429242 or vehicle control (dH2O) and analyzed on day 6 using Illumina bead arrays. (**B**) Selected genes showing greater than 1.4-fold change in expression are depicted. (**C**) Enrichment of gene signatures was determined using a hypergeometric test against a curated set of 28,000+ signatures and ontology term associations. Enriched signatures and ontologies were clustered according to genes contributing to enrichment with summary labels shown on the right.

The changes in the characteristics described above suggest that inhibition of S1P might impact on the gene expression programs governing PC differentiation or survival. To determine the potential role that S1P-regulated pathways might play in this process, RNA samples were prepared from cells that had been treated from day 3 for 72 h with PF-429242 or vehicle and processed for evaluation of gene expression. 439 genes were significantly upregulated and 404 genes significantly downregulated (FDR corrected p-value < 0.05 and fold-change >1.4) following treatment (Fig. 6A) (Supplementary Table S1). Genes upregulated after PF-429242 treatment included genes such as *EGR2*, *MYB*, *MIR155HG*, *SPIB*, *NFKBIE* and chemokines *CCL22* and *CCR7* associated with the activated B-cell state (Fig. 6B). In contrast genes downregulated following S1P inhibition included genes such as *SCD*, *ALDOC*, *LDLR*, *INSIG1*, *MSMO1* and *PFKFB4* which together comprise the primary components of the sterol biosynthesis pathway, as well as other metabolic genes. Notably, the expression of other important regulators of plasma cell differentiation, including *PRDM1* and *XBP1* was not altered (Supplementary Table S1). To assess differentially expressed genes systematically we applied an integrated gene signature and ontology analysis (Fig. 6C). This confirmed the significant association of genes which were upregulated following PF-429242 treatment with signatures of the activated B-cell state and NF-κB signaling, chemokine and cytokine signaling, and lysosomal components. In contrast, the signatures most significantly associated with genes down-regulated after PF-429242 treatment were related to SREBP controlled pathways and lipid metabolism, suggesting that a substantial proportion of the change in gene expression might be attributable to loss of SREBP processing and activation. However inhibition of S1P also affected genes linked to serine and amino acid synthesis and sets of genes linked to both signatures of mTOR signaling and regulation by IRF4. Collectively, these results are consistent with the impact of S1P inhibition on multiple linked transcriptional pathways, with CREB3L2 providing an indicator of sensitivity to S1P inhibition.

## Discussion

The UPR is required during the transition from activated B-cell to the ASC state and XBP1 is a key component of the conserved response to ER stress^10^. However, data suggests that early ASCs can still form in the absence of XBP1^2,6,7^ therefore posing the problem of how ASCs cope with their metabolic requirements prior to the expression of XBP1. These data suggest that additional arms of the UPR may be acting prior to or in conjunction with XBP1. It is also entirely conceiveable that different components of the UPR are engaged during the different phases of plasma cell generation. ATF6, the structurally homologous CREB3 family (including CREB3L2) and the sterol regulatory element binding proteins (SREBPs) can be regulated by intramembrane proteolysis during the UPR^25,26^. Additionally, direct regulation via alternative pathways has been identified. For example, TLR signals can trigger *XBP1* mRNA processing in the absence of an established UPR^27^, while insulin signalling can directly regulate the generation of processed SREBPs in specialised cell types^28^. Therefore transcription factor regulation linked to the UPR may also be controlled independently of ER stress.

Gene expression data has shown that *CREB3L2* increases as B-cells transition to ASCs, prompting the hypothesis that CREB3L2 may be a critical participant in adaptation to secretory load^15,18^. The generation of cleaved CREB3L2 coincides with immunoglobulin production and would fit with a response to ER stress. This pattern is similar to CREB3L2 expression in differentiating chondrocytes^29^. In this lineage, differentiation is accompanied by an increase in the production of cartilage matrix proteins, resulting in ER stress. In this setting, CREB3L2 is required for enhanced ER to Golgi trafficking and is essential for the generation of mature chondrocytes.

Accompanying the change in TF protein expression, application of PF-429242 had a profound effect on the biology of differentiating B-cells. Treatment of activated B-cells with this tool compound led to a dramatic reduction in cell number that was mirrored by an increase in autophagy. Interestingly, PF-429242 treatment did not preclude the development of phenotypic plasmablasts nor did the compound have substantial effects on plasmablasts during the subsequent progression to the PC state. Indeed, amongst the small number of cells retaining the capacity to differentiate after PF treatment, CD38 levels and CD138 percentages were increased. Given the loss of TF protein expression it is unlikely that such cells were displaying resistance to S1P-inhibition, but instead may have more effectively engaged in adaptive responses, such as autophagy, that allowed differentiation despite the impact of S1P inhibition on metabolic pathways.

After an initial activating stimulus coupled to growth, differentiating B-cells undergo multiple divisions linked to repeated stochastic cell fate choices between further division, ASC differentiation or death. During this process both the acquisition of CD38, particularly in the memory compartment, and class switch recombination are coupled to division number^30–32^. The data obtained with PF-429242 suggest that S1P-regulated pathways may alter the output during this phase of expansion. Overall the results showed a greater effect of PF-429242 on IgG secreting cells compared to IgM secreting cells. The explanation for this differential sensitivity potentially relates to origin from memory versus naïve B-cells, although a direct effect on class switching cannot be entirely excluded. Alternatively, specific differences in the nature of secretory pathway adaption linked to isotype may be involved. To our knowledge, differences separating the generation of IgG and IgM ASCs are not well characterised at present and this will be interesting to explore in future. The more pronounced loss of secretion in the CD38^low^ population, which have been postulated to represent short-lived ASCs, might be consistent with the idea that the level of plasma cell TFs affect the proportion of ASCs formed during each cell division^32^.

The gene expression data are consistent with the observed phenotypic and functional effects. S1P inhibition both delays the loss the markers of the activated B-cell state, and leads to impaired upregulation of a set of genes linked to a coherent set of genes connected to metabolic regulation. The genes which fail to be appropriately upregulated are linked to multiple aspects of metabolism, such as amino acid synthesis, steroid synthesis, and lipid/cholesterol biosynthetic processes. These data are in keeping with the prediction that S1P-regulated pathways might be altering the ability of differentiating cells to upregulate the necessary metabolic pathways essential for organelle expansion linked to increased secretory capacity, and the original targeting of the PF-429242 compound to the regulation of the SREBPs.

In summary, the data identify CREB3L2 as a transcription factor up-regulated during PC differentiation, are consistent with control through a pathway of intramembrane proteolysis which can be inhibited via targeting of S1P, and provide a sensitive indicator of effective S1P inhibition. S1P inhibition shows a striking dichotomoy in B-cell differentiation with clear sensitivity leading to profound effects on cell number and function during the activated B-cell to plasmablast transition, contrasting with resistance to inhibition as assessed by a range of parameters during the transition of plasmablasts to PCs. These results suggest that there are differences in the S1P-regulated processes at these closely linked phases of B-cell differentiation, with S1P regulated pathways potentially acting through the regulation of genes required for secretory pathway adaptation prior demands of immunoglobulin production. The results suggest that CREB3L2, most likely in conjunction with ATF6 and SREBPs, is involved in the early commitment to ASC fate.

## Materials and Methods

### Reagents

Reagents utilized in the *in vitro* culture system include human IL-2 (Roche); IFN-γ (Sigma); IL-6, IFN-α and IL-21 (PeproTech); goat anti-human F(ab’)_2_ fragments (anti-IgM and -IgG; Jackson Immunoresearch); HybridoMax hybridoma growth supplement (Gentaur); Lipid Mixture 1, chemically defined (200X) and MEM Amino Acids Solution (50X; Sigma); and PF-429242 (Tocris Bioscience).

The antibodies employed for protein detection include CREB3L2 (NBP1-88697, Novus Biologicals), ATF6 (40256; Novus Biological), ACTIN (A1978; Sigma), LC3A/B (Cell Signaling). The reagents utilized in flow cytometry assays include PE-conjugated anti-CD19 (LT19; Miltenyi Biotech), V450-conjugated anti-CD20 (2H7; E-Biosciences), PE-Cy7-conjugated anti-CD38 (HB7; BD Biosciences); APC-conjugated anti-CD138 (B-B4; Miltenyi Biotech); CountBright beads (Invitrogen) and 7-AAD (BD Biosciences).

### Cell culture

Whole blood obtained from healthy volunteer donors was collected in EDTA tubes after informed consent. Approval for this study was provided by U.K. National Research Ethics Service via the Leeds East Research Ethics Committee (approval reference: 07/Q1206/47). All the experimental protocols were conducted following standard procedures in compliance with the guidelines and regulations for Research Ethics Committees in the UK.

Peripheral blood mononuclear cells were isolated by density centrifugation followed by purification of total B-cells by negative selection using a memory B-cell isolation kit (Miltneyi Biotec). Procedures for the *in vitro* differentiation of B-cells have been described in detail elsewhere^15^.

### Protein evaluation

Cells were lysed by RIPA buffer (50 mM Tris pH 7.4, 150 mM NaCl, 1 mM EDTA, 1% NP40, 0.5% sodium deoxycholate, 0.1% SDS with the addition of protease inhibitors), loaded on to SDS-polyacrylamide gels and transferred to nitrocellulose membrane (Thermo Scientific). Membranes were probed with the appropriate antibodies and detected by Supersignal West Pico Chemiluminescent substrate (Thermo Scientific).

### Flow cytometry

Approximately 2 × 10^5^ cells per sample were stained with the indicated antibodies. Non-specific staining was blocked using buffer containing 10% BSA, human IgG (Sigma) and normal mouse serum (Sigma). Then, the cells were stained using directly conjugated isotype or antigen-specific antibodies. Live cells were gated using FSC/SSC characteristics and exclusion of 7-AAD (BD Biosciences). The phenotype was evaluated on an LSRIII flow cytometer (BD Biosciences) using DIVA software (BD Biosciences). Absolute cell counts were determined by CountBright bead assay (Invitrogen). Day 6 *in vitro* generated plasmablasts were labelled with anti-CD38 and 7-AAD prior to sorting on a BD Biosciences Influx.

### ELISA and ELISpot assays

Secreted immunoglobulins were detected using ELISAs performed as per the manufacturer’s instructions for the human IgM quatification kit (E80–100, Bethyl Laboratories, Inc) and human IgG kit (E80–104). Similarly, ELISpot kits for the detection of human IgM and IgG (Mabtech) were performed as described in the manufacturer’s protocol. Images were taken using an AID ELISpot Reader System (Autoimmun Diagnostika).

### Gene expression

RNA was extracted using TRIZol and subjected to DNAseI treatment (DNA Free, Ambion). RNA samples were amplified using Illumina Totalprep RNA Amplification Kit. Resulting cRNA samples were evaluated using whole-genome expression direct hybridization assay (HumanHT-12 v4 Expression BeadChips; Illumina). Data was processed as described previously^21^.

## Electronic supplementary material

Supplementary Table S1

## Data Availability

The complete set of array data has been submitted to the NCBI Gene Expression Omnibus (GEO; http://www.ncbi.nlm.nih.gov/geo) under accession number GSE118172.

## References

[1] Iwakoshi NN (2003). Plasma cell differentiation and the unfolded protein response intersect at the transcription factor XBP-1. Nat Immunol.

[2] Todd DJ, Lee AH, Glimcher LH (2008). The endoplasmic reticulum stress response in immunity and autoimmunity. Nat Rev Immunol.

[3] Reimold AM (2001). Plasma cell differentiation requires the transcription factor XBP-1. Nature.

[4] Hu CC, Dougan SK, McGehee AM, Love JC, Ploegh HL (2009). XBP-1 regulates signal transduction, transcription factors and bone marrow colonization in B cells. EMBO J.

[5] McGehee AM (2009). XBP-1-deficient plasmablasts show normal protein folding but altered glycosylation and lipid synthesis. J Immunol.

[6] Todd DJ (2009). XBP1 governs late events in plasma cell differentiation and is not required for antigen-specific memory B cell development. J Exp Med.

[7] Taubenheim N (2012). High rate of antibody secretion is not integral to plasma cell differentiation as revealed by XBP-1 deficiency. J Immunol.

[8] Gass JN, Jiang HY, Wek RC, Brewer JW (2008). The unfolded protein response of B-lymphocytes: PERK-independent development of antibody-secreting cells. Mol Immunol.

[9] Aragon IV, Barrington RA, Jackowski S, Mori K, Brewer JW (2012). The specialized unfolded protein response of B lymphocytes: ATF6alpha-independent development of antibody-secreting B cells. Mol Immunol.

[10] Gass JN, Gunn KE, Sriburi R, Brewer JW (2004). Stressed-out B cells? Plasma-cell differentiation and the unfolded protein response. Trends Immunol.

[11] Chan CP, Kok KH, Jin DY (2011). CREB3 subfamily transcription factors are not created equal: Recent insights from global analyses and animal models. Cell Biosci.

[12] Asada R, Kanemoto S, Kondo S, Saito A, Imaizumi K (2011). The signalling from endoplasmic reticulum-resident bZIP transcription factors involved in diverse cellular physiology. J Biochem.

[13] Lui WO (2008). CREB3L2-PPARgamma fusion mutation identifies a thyroid signaling pathway regulated by intramembrane proteolysis. Cancer research.

[14] Hawkins JL (2008). Pharmacologic inhibition of site 1 protease activity inhibits sterol regulatory element-binding protein processing and reduces lipogenic enzyme gene expression and lipid synthesis in cultured cells and experimental animals. J Pharmacol Exp Ther.

[15] Cocco M (2012). *In vitro* generation of long-lived human plasma cells. J Immunol.

[16] Jourdan M (2009). An *in vitro* model of differentiation of memory B cells into plasmablasts and plasma cells including detailed phenotypic and molecular characterization. Blood.

[17] Jourdan M (2014). IL-6 supports the generation of human long-lived plasma cells in combination with either APRIL or stromal cell-soluble factors. Leukemia.

[18] Shi W (2015). Transcriptional profiling of mouse B cell terminal differentiation defines a signature for antibody-secreting plasma cells. Nat Immunol.

[19] Xu H (2015). Regulation of bifurcating B cell trajectories by mutual antagonism between transcription factors IRF4 and IRF8. Nat Immunol.

[20] Carotta S (2014). The transcription factors IRF8 and PU.1 negatively regulate plasma cell differentiation. J Exp Med.

[21] Care MA (2016). Network Analysis Identifies Proinflammatory Plasma Cell Polarization for Secretion of ISG15 in Human Autoimmunity. J Immunol.

[22] Kondo S (2007). BBF2H7, a novel transmembrane bZIP transcription factor, is a new type of endoplasmic reticulum stress transducer. Mol Cell Biol.

[23] Care MA (2013). A Microarray Platform-Independent Classification Tool for Cell of Origin Class Allows Comparative Analysis of Gene Expression in Diffuse Large B-cell Lymphoma. Plos One.

[24] Turner ML, Hawkins ED, Hodgkin PD (2008). Quantitative regulation of B cell division destiny by signal strength. J Immunol.

[25] Brown MS, Ye J, Rawson RB, Goldstein JL (2000). Regulated intramembrane proteolysis: a control mechanism conserved from bacteria to humans. Cell.

[26] Lal M, Caplan M (2011). Regulated intramembrane proteolysis: signaling pathways and biological functions. Physiology (Bethesda).

[27] Martinon F, Chen X, Lee AH, Glimcher LH (2010). TLR activation of the transcription factor XBP1 regulates innate immune responses in macrophages. Nat Immunol.

[28] Shao W, Espenshade PJ (2012). Expanding roles for SREBP in metabolism. Cell Metab.

[29] Saito A (2009). Regulation of endoplasmic reticulum stress response by a BBF2H7-mediated Sec. 23a pathway is essential for chondrogenesis. Nat Cell Biol.

[30] Hasbold J, Corcoran LM, Tarlinton DM, Tangye SG, Hodgkin PD (2004). Evidence from the generation of immunoglobulin G-secreting cells that stochastic mechanisms regulate lymphocyte differentiation. Nat Immunol.

[31] Avery DT (2005). Increased expression of CD27 on activated human memory B cells correlates with their commitment to the plasma cell lineage. J Immunol.

[32] Tangye SG, Avery DT, Hodgkin PD (2003). A division-linked mechanism for the rapid generation of Ig-secreting cells from human memory B cells. J Immunol.

